# A study of the influence of measurement timescale on internal resistance characterisation methodologies for lithium-ion cells

**DOI:** 10.1038/s41598-017-18424-5

**Published:** 2018-01-08

**Authors:** Anup Barai, Kotub Uddin, W. D. Widanage, Andrew McGordon, Paul Jennings

**Affiliations:** 0000 0000 8809 1613 grid.7372.1 WMG, University of Warwick, Coventry, CV4 7AL United Kingdom

## Abstract

The power capability of a lithium ion battery is governed by its resistance, which changes with battery state such as temperature, state of charge, and state of health. Characterizing resistance, therefore, is integral in defining battery operational boundaries, estimating its performance and tracking its state of health. There are many techniques that have been employed for estimating the resistance of a battery, these include: using DC pulse current signals such as pulse power tests or Hybrid Pulse Power Characterization (HPPC) tests; using AC current signals, i.e., electrochemical impedance spectroscopy (EIS) and using pulse-multisine measurements. From existing literature, these techniques are perceived to yield differing values of resistance. In this work, we apply these techniques to 20 Ah LiFePO_4_/C_6_ pouch cells and use the results to compare the techniques. The results indicate that the computed resistance is strongly dependent on the timescales of the technique employed and that when timescales match, the resistances derived via different techniques align. Furthermore, given that EIS is a perturbative characterisation technique, employing a spectrum of perturbation frequencies, we show that the resistance estimated from any technique can be identified – to a high level of confidence – from EIS by matching their timescales.

## Introduction

Batteries play a significant part in powering modern technology, from consumer goods to electric vehicles and renewable energy storage systems^1^. It is important that the battery is able to safely, reliably, and efficiently provide and accept power and store energy as required. The performance and efficiency of a lithium ion battery is largely governed by the resistance of the electrochemical system. As the cell ages, through storage and cycling, this efficiency becomes progressively worse^2^. Knowing and understanding cell resistance therefore, is crucial in defining cell performance for different battery states and operating conditions. Internal resistance is also a critical index to define state of health (SoH) for lithium ion batteries^3^.

Cell resistance also has implications for the performance of the entire battery system. Battery systems in applications such as electric vehicles (EVs) employ a large number of cells connected in series and parallel. Unbalanced systems with differences in cell resistance limit the power delivery capability when connected in series. In parallel arrangements, significant differences in cell resistance result in non-uniform current loads in the pack^4^ leading to temperature gradients and consequently varying levels of cell degradation^1^.

In addition to thermal gradients across the battery pack, thermal gradients can also develop across individual cells, both along and normal to the electrode stack, due to inhomogeneous local current distributions under operational conditions, or internal manufacturing defects^5^. Such inhomogeneity results in localised heating, leading to local cell temperature ‘hot spots’ approaching values close to which the separator can melt leading to thermal runaway^6^. Internal defects giving rise to such local hot spots are correlated to localised film formation (SEI layer) and consequently localised discrepancies in resistance^7^.

Measurement techniques have traditionally employed either direct current (DC) or alternating current (AC) loads to calculate DC resistance (large current resistance) or AC resistance (small signal resistance) respectively^8,9^. In complex electrochemical systems such as a Li-ion battery, electrochemical processes, electrode microstructures and complex transport phenomena all contribute to internal resistance^10^. Furthermore, the state of the battery, namely: the battery’s state of charge (SoC)^11^, temperature^12^ and SoH affects the measured resistance^8^. Given the performance of Li-ion batteries depends on SoC, temperature and SoH, tests used to derive resistance are designed such that SoC, temperature and SoH remain unchanged^13–15^ during the course of testing. The value of resistance measured will thus depend on the remaining degree of freedom: the measurement duration (timescale) of the measurement, which is related to the underlying electrochemical process involved.

Pulse power tests usually have pulse lengths in the order of 1–30 seconds; at this timescale electron transfer, ion transfer and ion diffusion will contribute to resistance^16^. AC resistance on the other hand, historically, employed a sinusoidal AC signal of 1 kHz to measure the resistance^17^. At such large frequencies, depending on the particular battery technology, the cells will either be dominated by inductive or conductive behaviour. Electrochemical impedance spectroscopy (EIS) is a perturbative characterisation technique employing a spectra of perturbation frequencies which reveals the underlying electrochemical process across a wide frequency range^18^. The signal amplitude used in EIS is relatively very low compared to DC pulses, hence the resistance measured with this technique is sometimes known as the small signal resistance.

Recent work by Omar *et al*.^19^ indicates that in addition to SoC and temperature, current amplitude can also affect battery resistance. To account for current dependencies, the pulse power characterisation method uses a series of discharge and charge current pulses of increasing C-rates applied at pre-defined SoC and temperatures. In order to better represent the frequency bandwidth in application, Widanage *et al*.^20^ proposed a new signal design technique to generate a pulse-multisine signal, which is more dynamic in amplitude and frequency relative to standard pulse power tests and was shown to better predict battery performance, when subsequently applied to a model of a battery operating using real world duty cycles^21^.

While it may, naively, be expected that the internal resistance of a battery is the same irrespective of the technique employed, some authors have found that in practice resistance varies with the measurement technique used. Schweiger *et al*.^22^ attempted to categorise this in their study based on a 2 Ah cell. They calculated resistance using the pulse power method, EIS technique and Joule heating (thermal loss method). In the latter, heat measured using a calorimeter – under cycling – is entirely attributed to Joule heating; resistance is then calculated by equating the generated heat to i^2^R. In this technique, reversible entropic heat, side reactions and the heat of mixing^2^ are ignored which was shown to be important at low SoC^16^. Furthermore, this technique, which is employed in applications such as thermal management system design^23,24^, is complex, costly and the subsequent results have large uncertainty. This has restricted its widespread use for estimating internal resistance. More importantly, Schweiger *et al*. concluded that the AC impedance measured from EIS tests cannot be directly compared to that from pulse power test because of the complex electrochemical nature of the cell, without offering further analysis of the root cause. This may not necessarily be valid; there could be different underlying mechanisms contributing to the discrepancy between these techniques, and this has been investigated as part of this research. In recent work by Waag *et al*.^8^, the EIS technique and resistance measurements using a single charge-discharge pulse pair were applied to study changes in internal resistance as a function of SoC, temperature and current over the lifetime of a battery. A comparison of resistance calculated via the two techniques highlighted discrepancies, which the authors attributed to the non-linearity of the electrochemical device based on a theoretical understanding developed for lead-acid batteries^25^. Besides the work of Schweiger *et al*.^22^ and Waag *et al*.^8^, many other published works have used more than one technique for measuring resistance. For example, ageing studies^2,9,26^ have all used more than one technique for measuring resistance rise. Given that battery testing is costly, time consuming and can introduce unwanted ageing, characterisation testing should be minimised to a level where techniques that do not provide unique data are made redundant.

Since the work of Schweiger *et al*., existing techniques for resistance measurements have been updated and new techniques such as the pulse-multisine method have been proposed. Any up-to-date, rigorous analysis of these methods, therefore, is also of value to the research community. The consistency of the techniques is vital for future ageing studies; given that EIS and pulse current tests are often used together, being able to compare these complementary techniques accurately is important.

The primary objective of the aforementioned studies was not concerned with the techniques used to measure battery resistance and therefore did not consider the differences, or similarities, between the techniques themselves and the physical meaning of the resulting resistance measurements. In this work therefore, a subset of established techniques is presented. The various processes contributing to cell resistance are derived and explained in detail for each technique. Each technique is then applied to 20 Ah LiFePO_4_/C_6_ pouch cells and the results are used as a basis of comparison between the different techniques. It is shown that the timescales of the measurement technique govern the resulting resistance estimate. Consequently, it is shown that resistance derived from any technique can be estimated purely from the EIS data. Given that the EIS technique readily attributes timescales to physical processes, it is argued that the EIS technique may be a sufficient test for determining the battery resistance without requiring further tests.

The theoretical background of different methodologies for resistance measurements is outlined in the next section. Afterwards, a test matrix is proposed to measure resistance of cells by employing all of the techniques identified and results are presented along with the discussion. Finally, the overall contributions of this research is summarised.

## Theory of measuring resistance employing DC signals

**Figure 1:**
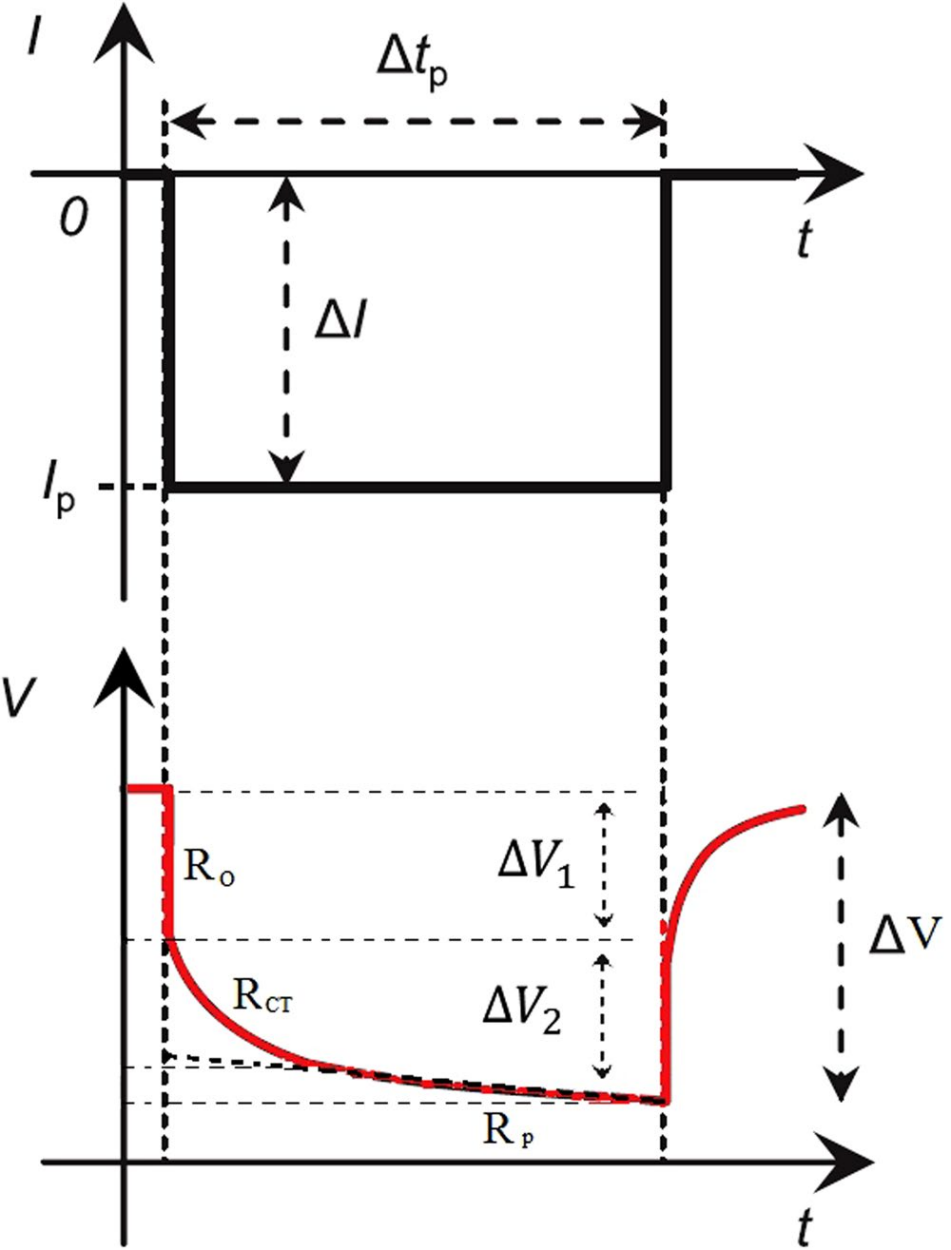
Cell voltage response to a pulse current.

The DC resistance of a battery is simply the ratio of voltage to current, arising from a given current/voltage perturbation (∆V/∆I). An example of voltage drop due to a step-current discharge pulse is shown in Fig. 1. There are a number of phenomena contributing to the voltage drop, governed by their respective timescales: (i) the instantaneous voltage drop is due to the pure Ohmic resistance R_0_ which comprises all electronic resistances and the bulk electrolyte ionic resistance of the battery^16^, (ii) the voltage drop within the first few seconds is due to the battery’s double layer capacitance and charge transfer resistance R_CT_ which is attributed to the charge transfer reaction at the electrode/electrolyte interface^8^, and (iii) the shallow, linear (or close to linear) voltage drop is due to polarisation resistance R_p_ which accounts for ionic diffusion in the solid phase and is usually considered to be the rate determining step for Li ion batteries^27^. The contribution of these three parts can be calculated separately for an intuitive understanding of the complex electrochemical processes involved in the battery system. On the other hand, a bulk total cell resistance can be calculated from the total voltage drop for the pulse, as is often done in the literature^15,28^. The other drawback of using DC current to obtain resistances is that only the imposition of all the different contributions to resistance, hence there in an inability to completely separate the different resistance components.

Internal cell resistance calculated from multiple discharge/charge pulses of different amplitudes is also common in literature and standards^13,29^. In this case, current pulses of different pre-defined amplitudes give rise to analogous voltage responses; resistance is then defined as the gradient of the voltage versus current plot. As long as the change in SoC is negligible and the battery does not enter into a regime of diffusion limitation^28^, the voltage response will remain linear. Furthermore, depending on how long into the pulse the voltage is measured, the gradient will represent a phenomenon between the pure Ohmic resistance (milliseconds) and the cell’s bulk total resistance (seconds). Therefore, as long as the current amplitude is not very high or very low (the definition of which is cell dependent), the resistance calculated using this method is expected to be similar to that calculated using a single pulse.

The pure Ohmic resistance R_0_ can be calculated from the falling edge of a voltage response pulse, from the instantaneous voltage drop when current stops. The value of R_0_can also be calculated from switching current, by measuring instantaneous voltage change due to any current change. In principle, the R_0_ value calculated with these methods should be equal to that calculated from the rising edge of the pulse. However, due to the preceding current load, in the falling edge scenario, the electrode surface of the cell is highly polarised^30^. When the current load is switched-off, the system equilibrates and the non-intercalated cations in the double-layer diffuse back into the electrolyte bulk. The difference in Li-ion concentration at the electrode/electrolyte interface between the rising and falling edge of a pulse, results in a small voltage differences and consequently R_0_.

## Theory of measuring resistance employing AC signal

Measuring battery resistance with a 1 kHz AC signal (or similar single frequency signal), is common practice in industry, especially for measuring lead-acid battery resistance. It is a relatively fast (in the order of seconds), low power consuming and low cost technique, using handheld equipment. Usually a low current sinusoidal signal of 1 kHz is applied to the battery and the voltage response is measured. Although this technique is time-efficient, a single value of resistance is not sufficient to characterise the battery’s performance. This is because charge transfer through multilayer surface films and kinetic and diffusional processes in the solid and liquid phases of the battery lead to a frequency dependent resistance. In equivalent circuit models, this frequency dependence is analogically represented by multiple resistance elements coupled with surface layer capacitances^12,31^. Therefore, a complete characterisation of battery resistance requires measurements spanning low (<1 Hz) to high (>100 kHz) frequencies.

Electrical impedance spectroscopy (EIS) employs multiple frequency sine waves to measure resistance over a wide range of frequencies. For EIS measurements, a frequency range of 100 kHz to 10 mHz is common in the literature^8,12,18^. The detailed theory of EIS has previously been discussed in the open literature^8,32^. In essence, a current/voltage signal (galvanostatic/potentiostatic) is applied and the voltage/current response is measured. From the voltage/current response, resistance is calculated.

Consider the galvanostatic excitation signal **i**(**t**)

1 $$i(t)={i}_{o}sin(\omega t+{\phi }_{1})$$

where **i**_**o**_ is the excitation magnitude, **ω** is the excitation frequency, **t** is time and **φ**_1_ is a phase angle. The resulting voltage response **v**(**t**) will have the same frequency, but different phase angle **φ**_2_:

2 $$v(t)={v}_{o}sin(\omega t+{\phi }_{2})$$

where **v**_***o***_ is the voltage amplitude. Eqs (3) and (4) is a simple mapping from time domain to phasor domain for a sinusoidal variable.

3 $$I(\omega )={I}_{o}(\omega ){e}^{j{\phi }_{1}(\omega )}$$

4 $$V(\omega )={V}_{o}(\omega ){e}^{j{\phi }_{2}(\omega )}$$

where j is the imaginary unit. The complex resistance as a function of frequency is then given by:

5 $$Z(\omega )=\frac{V(\omega )}{I(\omega )}\frac{{V}_{o}{e}^{j{\phi }_{2}}}{{I}_{o}{e}^{j{\phi }_{1}}}={Z}_{0}{e}^{j({\phi }_{2}-{\phi }_{1})}.$$

**Figure 2:**
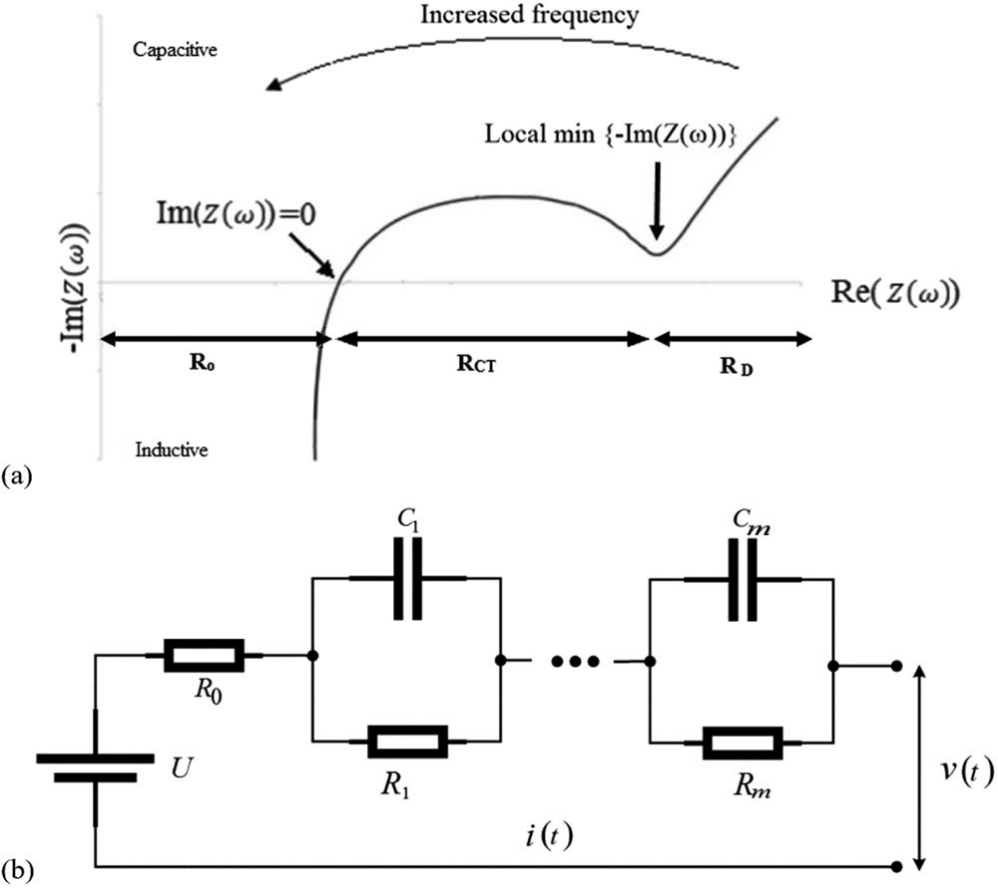
Typical Nyquist plot of a new li-ion battery cell (**a**), and an m^th^ order ECM (**b**). The series resistor R_0_ denotes the internal resistance and the m RC pairs denote the dynamic model parameters.

The Euler representation is

6 $$Z(\omega )={Z}_{o}({\cos }(\phi )+i\cdot \,{\sin }(\phi ))$$

where, **φ** = **φ**_2_ − **φ**_1_. The complex resistance **Z**(**ω**) has two parts: real and imaginary, which is commonly illustrated by a Nyquist plot. An example of an EIS Nyquist plot is shown in Fig. 2.

In the Nyquist plot, negative values of −Im(Z(w)) indicate inductive dominated behaviour (high frequency) while positive values of −Im(Z(w)) indicate capacitive dominated behaviour (mid-low frequency). At the point where Im(Z(ω)) = 0 both capacitive and inductive behaviours are balanced; this point is typically correlated with the pure Ohmic resistance R_0_^8^. Towards the low frequency region of the Nyquist plot, the local minimum of −Im(Z(ω)) can be found as indicated in Fig. 2(a). The difference between the Re(Z(ω)) at ${\rm{\min }}\{-{\rm{Im}}({\rm{Z}}({\rm{\omega }}))\}\,\,$and R_0_ corresponds to the charge transfer resistance R_CT_. The real part of Z(ω) at ${\rm{\min }}\{-{\rm{Im}}({\rm{Z}}({\rm{\omega }}))\},\,\,$in theory therefore, should correspond to the total resistance measured from other methods e.g. pulse power test.

Depending on cell type and experimental setup (e.g. cable assembly), the 1 kHz resistance measurement can be in the inductive or conductive region. Typically, for cells with large capacities (e.g. a 40 Ah pouch cell) the resistance at 1 kHz is dominated by inductive behaviour. On the other hand, cells with relatively lower capacities (e.g. a 3Ah 18650 cell) can have a 1 kHz resistance close to the point where Im(Z(ω)) = 0 ^29^, and thus give reliable and repeatable measurements.

## Theory of measuring resistance employing pulse-multisine signals

The pulse-multisine procedure^20^ is a method designed to better represent the frequency bandwidth of the current load in application than a pulse power test. It involves three steps in estimating the internal resistance of a battery. The first step is the design of a pulse-multisine signal, followed by estimating the resistance of the battery as a function of frequency and the third step is fitting an equivalent circuit model (ECM) to the resistance estimate from which the internal resistance is obtained.

A pulse-multisine signal is set up to span the full applicable maximum 10 seconds charge and discharge C-rates that a battery can support at a given temperature and SoC, while exciting the battery over a frequency range representing the realistic usage case. The designed pulse-multisine is then applied to the battery to estimate its resistance response as a function of frequency and subsequently the internal resistance.

The resistance **Z**(**ω**) at a given angular frequency **ω** is related to the complex current and voltage signal as

7 $$V(\omega )=Z(\omega )I(\omega )+E(\omega )$$

where **E**(**ω**) is the complex error that can arise due to any measurement error or nonlinear response of the battery. To estimate the resistance **Z**(**ω**)in Equation (7) given **I**(**ω**)and **V**(**ω**)requires minimising the complex error in a least squares sense. A method known as the Local Polynomial Method^21^ is used to estimate the resistance $\hat{{\bf{Z}}}({{\boldsymbol{\omega }}}_{{\bf{k}}})$ along with its standard deviation ${{\boldsymbol{\sigma }}}_{\hat{{\bf{Z}}}}({{\boldsymbol{\omega }}}_{{\bf{k}}})$.

Once the non-parametric resistance ($\hat{{\bf{Z}}}({{\boldsymbol{\omega }}}_{{\bf{k}}})$) is estimated, an ECM model is fitted to it to obtain the internal resistance along with other dynamical parameters. A general m^th^ order ECM is shown in Fig. 2(b). The internal resistance of interest is denoted by the series resistance R_0_ while the remaining RC parameters denote the polarisation dynamics and the open circuit voltage **U**.

The resistance of this general m^th^ order ECM is given in Equation (8).

8 $${Z}_{m}(\omega )={R}_{0}+\sum _{i=1}^{m}\frac{{R}_{i}}{j\omega {\tau }_{i}+1}$$

The first term **R**_0_ is the pure resistive resistance and the remaining m-terms correspond to the resistance of the **m** RC pairs with the product **R**_**i**_ **C**_**i**_ denoting the time constant **τ**_**i**_ of the **i** ^**th**^ RC pair.

To estimate the parameters **R**_0_, **R**_**i**_ and **τ**, the model order m should be decided in advance. The estimated impedance response can support the choice of the model order^21^, however, in the absence of resistance response, typical model orders are first or second order^27,33^. In this manuscript, a second order model is considered to estimate the parameters as this has been shown to capture cell behaviour most accurately^21^.

## Experimental Method

In this work, commercially available 20 Ah pouch cells with a graphite (LiC_6_) negative electrode and lithium iron phosphate (LiFePO_4_) positive electrode are used. The maximum charge voltage for the cells is 3.6 V (3.8 V for 10 sec pulse current) and minimum discharge voltage is 2.0 V (1.6 V for 10 sec pulse current). The manufacturer defined maximum charge and discharge capability are 15 C instantaneous. All the tests as outlined below were performed on each cell.

At the beginning of testing, the SoC for each of the cells was adjusted to 50% at 25 °C, using a commercial cell cycler (Bitrode MCV 16-100-5) and an environmental chamber (Weiss Gallenkamp Votsch VC^3^ 4060). The adjustment protocol includes discharging the cells to the manufacturer defined minimum discharge voltage (defined as 0% SoC), which is followed by a 4 hour rest period. Subsequently the cells are charged using a constant current – constant voltage protocol (CC-CV) using the 1 C current rate for the CC part until 3.6 V is reached and then holding the cells at 3.6 V for the CV part, until the current falls below the C/20 cut-off current. After a 4 hour rest period, the cells discharged at the 1 C rate for 30 min to adjust to 50% SoC. Another 4 hour rest period was applied, allowing the cells to reach electrochemical equilibrium^18^. The five internal resistance estimation methods were then applied to characterise the resistance at 50% SoC at 25 °C.

The pulse power test with a single discharge/charge pulse was applied at 5 C current for 18 seconds and the cells rested for an hour prior to charging at 5 C for 18 seconds. The pulse duration of 18 seconds was chosen because it is one of the longest pulse durations outlined in current standards^14^.

The next test performed on the cells was the pulse power test with multiple pulses as suggested in ref.^13^. In this test, cells were charged and discharged with 10 second pulses at 1 C, 2 C, 5 C and maximum C, with intermediate 30-minute rest steps after each pulse. The individual pulses can be used to calculate resistance as per the methodology described in Section 2.1.

In the switching current methodology the current is step changed (here 1 C to 5 C are used as suitable low and medium current amplitudes) and the voltage change due to this current step change is measured; internal resistance is then calculated via Ohm’s law^22^. The current can be changed during discharge, charge or from discharge to charge. For the latter, the current is switched, in this work, from 5 C discharge to 5 C charge (both with 5 second pulses). In the discharge only case, the current is switched from −1C to −5C during discharge, and finally in the charge only case, the current is switched from 1 C to 5 C during charge. Here, 1 C and 5 C current values are used as representative only.

Following resistance measurements using DC current pulses, resistance was measured with AC current signals. The 1 kHz resistance was measured with a Hioki BT3563 1 kHz resistance tester at 50% SoC, 25 °C.

Galvanostatic EIS tests were performed using a Solartron Modulab system (model 2100 A) fitted with a 2 A booster card. Multiple EIS measurements were taken on the same cell under the same test conditions within the frequency range of 10 mHz to 100 kHz, using different RMS current values: 0.2 A, 0.5 A, 0.8 A, 1.0 A and 1.4 A. These current values were selected such that they were not too high to change the cell’s SoC during measurement but high enough for a good signal to noise ratio for the response voltage signal. This was to analyse the EIS dependency on the amplitude of the signal current.

With the pulse-multisine signal procedure, five periods of the signal were applied, with the battery adjusted to 50% SoC and allowed to equilibrate, and the corresponding voltage response recorded. Once the data was collected, the non-parametric resistance and ECM were fitted as described in previous Section.

### Results of the experiments employing DC signals

**Figure 3:**
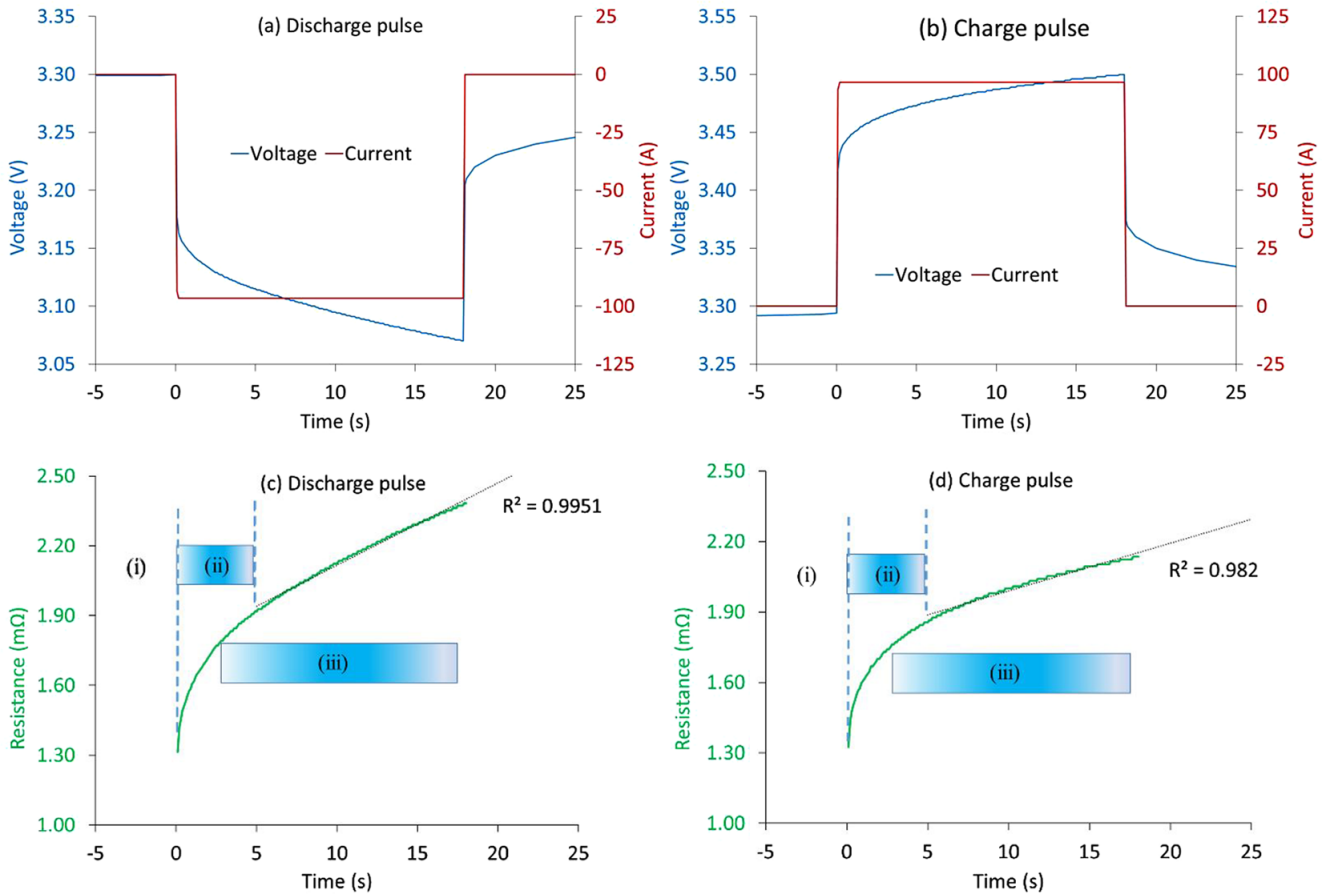
Voltage response to 18 second (**a**) discharge pulse and (**b**) charge pulse. Resistance calculated for pulse duration are shown in (**c**) for discharge and (**d**) for charge. In subplots (**c**) and (**d**), (i), (ii) and (iii) refer to pure Ohmic resistance R_0_, charge transfer resistance R_CT_ and polarization resistance R_p_, respectively. The overlap between (ii) and (iii) is indicative of the inability, within this technique, of precisely discerning each contribution.

Pulse power test results with a single 5 C discharge and charge pulse of 18 sec are shown in Fig. 3. Using this pulse, the DC resistance can be calculated for any length of pulse up to 18 seconds. The pure Ohmic resistance was calculated from the voltage drop after 0.1 sec of the pulse current. Ideally, the pure Ohmic resistance should be calculated from the instantaneous drop of voltage due to change in current. However, measurement of the instantaneous drop is limited by the data acquisition rate of the equipment used. For this experiment, the battery cycler that was used has a maximum resolution of 0.1 sec.

Since the electrodes are thin (as it is a high power cell) and the electron pathways for charging and discharging are similar in the cells used, the pure Ohmic resistances (resistance calculated at 0.1 sec after the onset of the pulse) are comparable as shown in Table 1. The longer-time resistance for charge and discharge however, are different as illustrated in Fig. 3(c,d), and Table 1. During charge, the positive electrode material is oxidised, Li ions are de-intercalated from the layered lithium intercalation host, in this case LiFePO_4_, pass across the electrolyte and are intercalated between the graphite layers by an electrochemical reduction reaction proceeding at the negative electrode. On the other hand, during discharge, an oxidation reaction occurs at the negative electrode, Li ions are de-intercalated from the anode, and migrate across the electrolyte to be re-intercalated into the positive electrode material, where a simultaneous electrochemical reduction reaction proceeds. In general, the higher the electrode potential, the harder it is to remove a lithium from a site within the host matrix. On discharging a cell, Lithium is transferred from a high energy state in the anode to a low energy configuration in the cathode, hence, the resistance values for discharging are higher than that of charging at 50% SoC; this has also been found by other researchers^8,22^.

**Table 1:** Change of internal resistance with pulse duration.

Pulse Duration (s)	Discharge Pulse (mΩ)	% Change	Charge Pulse (mΩ)	% Change	Breakdown of resistance
0.1	1.31 ± 0.02	—	1.32	—	R_O_ + %R_CT_
2	1.72 ± 0.02	31%	1.70	29%	R_O_ + R_CT_
5	1.92 ± 0.02	46%	1.85	41%	R_O_ + R_CT_ + %R_P_
10	2.12 ± 0.02	62%	2.00	52%	R_O_ + R_CT_ + R_P_
18	2.38 ± 0.02	82%	2.13	62%	R_O_ + R_CT_ + R_P_

The resistance in Fig. 3(c) and (d) can be categorised into three parts associated with the processes contributing to the voltage drop discussed in Sec. 2.1., namely: (i) pure Ohmic resistance R_0_ resulting in the instantaneous voltage drop and is dominant up to 0.1 seconds, (ii) the charge transfer resistance R_CT_ occurring from circa. instantaneous up to 2–5 seconds and (iii) the slow, linear, solid phase lithium ion diffusion which inevitably results in concentration polarization R_p_, especially during high current charging, which takes the battery voltage rapidly up to the upper voltage limit, occurring at timescales 5 seconds^34^. Although R_o_, R_CT_ and R_p_ are not completely separated, at their respective timescales they are expected to be the dominant contribution to the total resistance.

The internal resistance calculated from five charge-discharge pulses of varying amplitudes is shown in Table 2. The 5 C test data is the same data as that shown in Table 1. The pure Ohmic resistance (calculated from the 0.1 sec voltage drop) remains similar for all pulse amplitudes, with a standard deviation of 0.05 mΩ. However, the difference in resistance value that was calculated at the end of 2 seconds, 5 seconds and 10 seconds varies with charge-discharge rates. For example, the difference between resistance measured with a 2 second and 10 sec pulse is 0.73 mΩ using a 1 C discharge pulse, while it is 0.39 mΩ using a 15 C discharge pulse. The discrepancies in the rate of resistance rise arising due to pulse amplitudes are attributed to the various electrochemical processes that are activated within the cells as the duration of the pulse is extended and the heat generation associated with pulse currents. For higher current pulses, such electrochemical processes are activated earlier (as the double layer can be discharged much quicker) – the large magnitude current values also cause the ratio V/I to be suppressed^8^. In addition, at higher rates, more heat is generated i.e. 0.5 Wh (1800 Joules) in just 10 seconds when 300 A (15 C) current is passed through a 2mΩ resistance, which effectively increases the internal temperature of the battery which contributes to the resistance decrease as seen in Table 2.

**Table 2:** Internal resistance (mΩ) calculated from different amplitude discharge pulses.

Pulse Length (s)	Internal Resistance (mΩ) for varying Rates			
	1 C	2 C	5 C	15 C
**From Discharge Pulse**				
0.1	1.35 ± 0.05	1.37 ± 0.03	1.31 ± 0.02	1.30 ± 0.01
2	1.76 ± 0.05	1.81 ± 0.03	1.72 ± 0.02	1.66 ± 0.01
5	2.07 ± 0.05	2.12 ± 0.03	1.92 ± 0.02	1.84 ± 0.01
10	2.49 ± 0.05	2.49 ± 0.03	2.12 ± 0.02	2.05 ± 0.01
**From Charge Pulse**				
0.1	1.35 ± 0.05	1.35 ± 0.03	1.32 ± 0.02	1.30 ± 0.02
2	1.76 ± 0.05	1.76 ± 0.03	1.70 ± 0.02	1.51 ± 0.02
5	2.02 ± 0.05	1.99 ± 0.03	1.85 ± 0.02	1.59 ± 0.02
10	2.33 ± 0.05	2.23 ± 0.03	2.00 ± 0.02	1.70 ± 0.02

For long duration pulses at higher rates, it is anticipated that changes in resistance will be dominated by changes in SoC. At the 1 C pulse rate, after 10 seconds, the SoC changes by an insignificant 0.28%; at the 15 C pulse rate, after 10 seconds, the SoC changes by 4.2% - which can lead to an appreciable voltage drop/rise. For the LiFePO_4_ battery the voltage plateau between 70% and 40% SoC^35^, where measurements for this work were taken, means that a 4.2% change in SoC has little impact. For battery technologies with steeper OCV curves, such as LiNiCoAlO_2_ and LiNiMnCoO_2_ however, the effect is expected to be more pronounced.

The results of Table 2 show distinctive peaks for resistance at 2 C for discharging and 1 C for charging for this method. Within the Butler-Volmer framework, these peaks may be associated with the temperature-overpotential duality.

Pure Ohmic resistance was also calculated from the falling/rising (charge/discharge) edge, at the end of a pulse. The pure Ohmic resistance calculated from 1 C, 2 C, 5 C and 15 C discharge pulses are 1.30 mΩ, 1.35 mΩ, 1.35 mΩ and 1.40 mΩ respectively and for 1 C, 2 C, 5 C and 15 C charge pulses are 1.40 mΩ, 1.40 mΩ, 1.40 mΩ and 1.56 mΩ respectively. On average, the values for discharge are less than 0.1 mΩ higher than those shown in Table 2. This may be related to the energy required for de-intercalation from the positive electrode and intercalation into the negative electrode being different from de-intercalation from the negative electrode and intercalation into the positive electrode.

**Figure 4:**
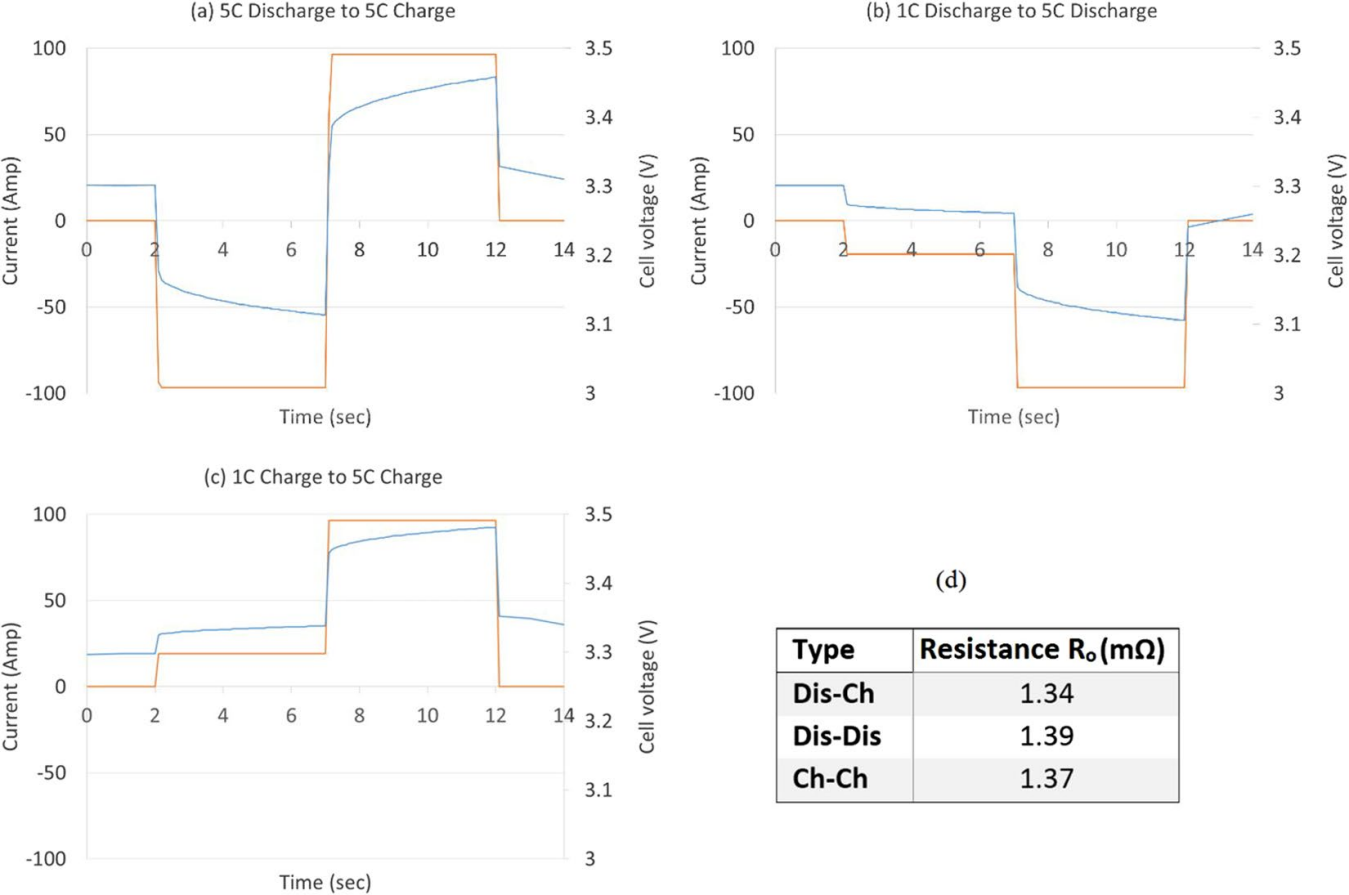
Voltage response to switching current from (**a**) 5 C discharge to 5 C charge, (**b**) 1 C discharge to 5 C discharge, (**c**) 1 C charge to 5 C charge, and (**d**) pure Ohmic resistance calculated from switching edge.

The voltage responses to the change of current from discharge to charge and current magnitudes during charge and discharge are shown in Fig. 4(a,b and c), respectively, Fig. 4(d) presents the pure Ohmic resistance calculated from the switching edge of a pulse current. The resistance calculated from changing the discharge current from 1 C to 5 C is similar to that calculated from the 0.1 second pulse and the falling edge method. In the charging scenario, current is switched from 1 C to 5 C, the resistance closely matches that calculated using the pulse power method. The resistance calculated by switching from discharge to charge closely matches the pure Ohmic resistance for charge current shown in Table 1 and Table 2.

### Results of the experiments employing AC signal

**Figure 5:**
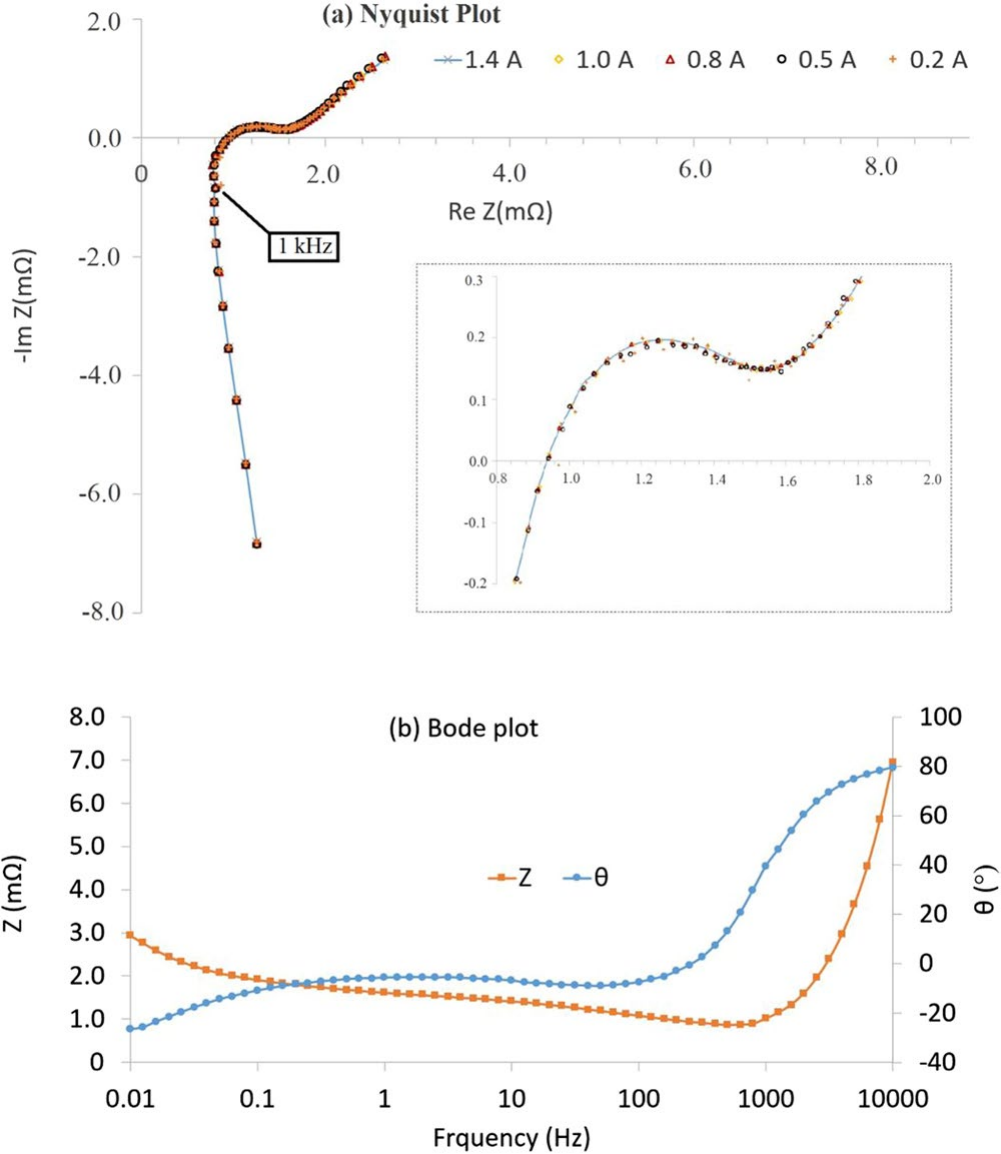
EIS results from 100 kHz to 10 mHz with different signal amplitudes (**a**) Nyquist plot and (**b**) Bode plot of same data. Inset (**a**) a zoomed view of the central part is shown.

The 1 kHz resistance measured using the Hioki 1 kHz resistance tester was 0.82 mΩ. The 1 kHz resistance, for these cells, lies in the inductive dominated region as can be seen in the EIS Nyquist plot in Fig. 5.

The EIS test results with different current amplitudes are presented in the form of a Nyquist plot in Fig. 5(a). There are no identifiable differences between results due to changing the amplitude of the galvanostatic signal, as expected. The zoomed view shown in the inset, shows slightly noisy results for 0.2 A which is supressed for 0.5 A and beyond. Therefore, resistance measured using EIS is not dependent on current amplitude, but using higher currents can reduce noise. Indeed, if a higher current like 1 C was used for the EIS test, it would have an effect on results, however application of such high current for EIS test has a little precedent in literature. A current amplitude of C/20 (in this case 1 A) can produce the same results repeatedly with low measurement noise, limited by the equipment sensitivity. The EIS results for the 1 A magnitude is presented as a Bode plot in Fig. 5(b).

From inspection of the Nyquist plot, the pure Ohmic resistance R_o_, was found to be 0.92 mΩ corresponding to 251 Hz (i.e., a timescale of 4ms). The resistance at the local minimum before the cells enters into the low frequency diffusion dominated region was found to be 1.55 mΩ, corresponding to 2 Hz; and thus R_CT_ is 0.63 mΩ. Estimating polarisation resistance using EIS results is not well-defined. Given that R_p_values for the DC pulse in this discussion was derived from a 10 second pulse, the 0.1 Hz result is used to define R_p_. The equivalent resistance at 0.1 Hz from EIS results corresponds to 0.36 mΩ; this becomes 1.39 mΩ when a frequency of 0.01 Hz is considered to calculate the R_p_ value.

### Results of the experiments employing a Multisine signal

**Figure 6:**
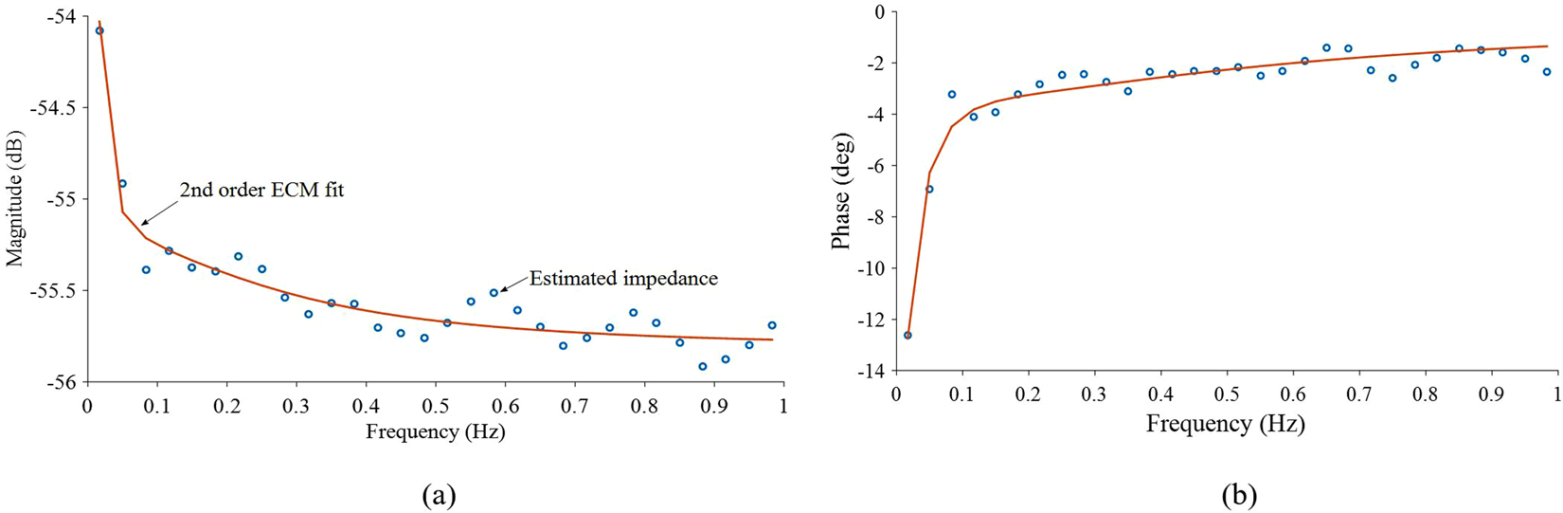
(**a**) Magnitude and (**b**) phase response of the estimated resistance via the LPM using a pulse-multisine signal and a fitted 2^nd^ order ECM model.

The phase and magnitude of the estimated resistance, based on the local polynomial method (LPM), and a 2^nd^ order ECM model fit are shown in Fig. 6. Although the ECM parameters are not uniquely identifiable, the series connected resistance and the resistance of the 1^st^ and 2^nd^ RC branches of the ECM are typically attributed to R_o_, R_CT_ and R_p_ which were found to be 1.618 ± 0.003 mΩ, 1.10 ± 0.07 mΩ and 0.109 ± 0.005 mΩ, respectively. The value of R_o_ is significantly higher than that of found with other techniques. This can be related to two potential reasons: i) the fact that most of the power in the driving current signal belongs to harmonics lower than 1 Hz, and/or ii) despite the good fit between model and experimental data, the parameters do not reflect the physical meanings attributed to them (due to unique identifiability).

### A comparison of the various resistance measurement techniques

The average pure Ohmic resistance values calculated using different pulse power techniques is 1.33 mΩ with standard deviation of 0.04 mΩ. All these measurements were taken 0.1 sec after the current pulse was applied. Irrespective of charge or discharge, the pure Ohmic resistance values measured by different DC techniques closely match, with a 3% variation, regardless of whether they were measured from the beginning, end or switching point of the pulse current.

**Figure 7:**
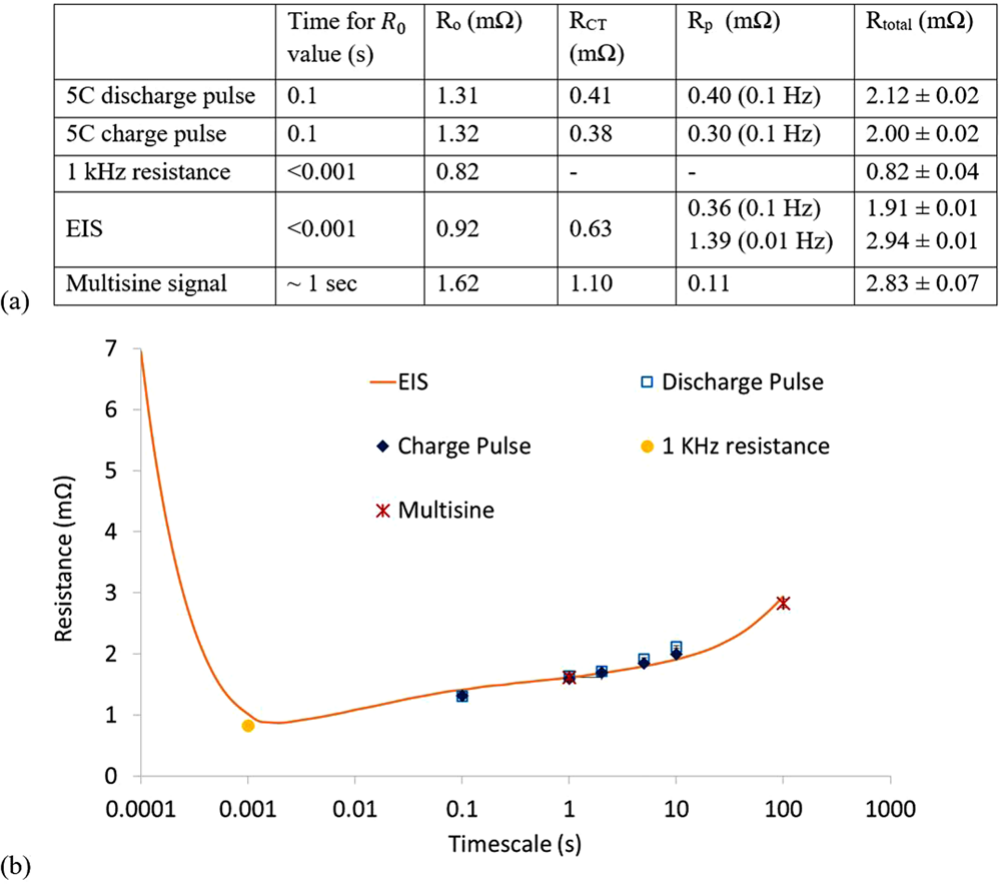
(**a**) Comparison of resistance values measured by different techniques. (**b**) Total resistance vs timescale plot showing resistance values agree when timescales match, irrespective to the measurement technique employed.

A comparison of R_o_, R_CT_ and R_p_ estimated using the techniques discussed above are presented in Fig. 7 (a). The sources of discrepancies between resistance values measured using charge and discharge pulses were discussed previously. The R_CT_ and R_p_ values are calculated using the 2 sec and 10 sec data points of the pulses.

The R_0_ value of 0.92 mΩ identified from EIS tests (Fig. 5) corresponds to the 251 Hz (4mS) frequency response; likewise, the R_CT_ value of 0.63 mΩ corresponds to the 2 Hz response. Unlike R_0_ and R_CT_ the polarisation resistance R_p_ isn’t identified from a Nyquist plot, instead it is pre-defined. In this study two frequencies were considered, 0.1 Hz and 0.01 Hz which gives values of 0.36 mΩ and 1.39 mΩ, respectively. Since the values of resistance R_o_, R_CT_ and R_p_ have physical meaning^36^, it is appropriate to define the timescales of R_o_, R_CT_ and R_p_ using EIS results.

Resistance measured using a 1 kHz signal lies in the inductive region of the Nyquist plot, see Fig. 5(a), which is close to the crossover point of the horizontal-axis. Therefore, it is reasonable that the 1 kHz resistance (0.82 mΩ) close to the Ohmic resistance identified from EIS test results (0.92 mΩ at 251 Hz, 0.79 mΩ at 1 kHz). It is worth reiterating that this may not be true for other batteries and the 1 kHz point can be well into the capacitive region e.g., for smaller pouch and cylindrical cells. This is particularly likely for smaller capacity cells, in which case the value will not solely represent the pure Ohmic resistance.

The average pure Ohmic resistance R_0_ found using DC methods of 1.315 mΩ, is much higher than that found using EIS. In pulse power tests, R_0_ is typically calculated using the 0.1 sec data point – i.e., it is governed by the lowest resolution of commercial battery cyclers today which is 10 Hz –whereas by definition it should be the instantaneous drop of voltage at the onset of current. Therefore, the DC resistance calculated from pulse power tests will contain kinetic contributions (a portion of R_CT_), pulling up/down the voltage response beyond the pure Ohmic contribution occurring at timescales of less than milliseconds. Since EIS results of this battery suggests that R_0_ corresponds to the 251 Hz (4mS) frequency response; it is therefore physically more meaningful to use the voltage drop after 4ms from the onset of a current pulse to determine R_0_. Although a square wave will excite frequencies beyond the 10 Hz frequency, a 0.1 sec pulse will have the highest harmonic contribution from the 10 Hz sine wave, the extracted values for R_0_ from DC pulse power tests and EIS tests therefore, will be less divergent. Given the limitation of existing commercial battery cyclers however, this is not feasible and can only be achieved when battery test equipment capable of reaching high pulse currents (from 0 Amp) within 4ms are available. By way of comparison, from Fig. 5(b), the resistance value at 10 Hz using the EIS technique was found to be 1.41 mΩ, which is comparable to the 1.33 mΩ estimated via DC pulse. Hence, the discrepancy in pure Ohmic resistance measured by the DC method can be attributed to limitations of the battery test equipment typically used.

Given that R_o_ measured by DC pulse will contain charge-transfer contributions, comparing R_CT_ values measured via EIS and pulse current techniques will have foreseeable differences. Similarly, R_CT_ measured using DC techniques will contain polarisation effects which are difficult to isolate. Nevertheless, here, we follow the usual prescription of calculating R_o_, R_CT_ and R_p_ with DC methods^8^. Using the 5 C pulse, considering 2 sec pulse duration, R_CT_ is estimated to be 0.41 mΩ and 0.38 mΩ for discharge and charge respectively. Due to the fact that a portion of R_CT_ is embedded into R_o_ (measured from 0.1 sec pulse duration), a comparison of R_CT_ values measured from pulse power test and EIS test in not meaningful. However, the R_o_ + R_CT_ value, measured after 2 sec pulse duration should be in close agreement with the resistance value deduced from a 0.5 Hz sine wave (as a 2 sec square wave has highest harmonic contribution from 0.5 Hz). From Fig. 5(b), the resistance value measured by EIS at 0.5 Hz is 1.69 mΩ, whereas the R_o_ + R_CT_ value measured from 2 sec pulse duration is 1.71 ± 0.01 mΩ, which are in close agreement.

Similarly, using a 5 C pulse, considering a 10 sec pulse duration, R_p_ values are estimated to be 0.40 mΩ and 0.30 mΩ for discharge and charge pulses respectively. Similarly to the case of R_CT_, R_p_ values from 10 sec pulse duration cannot be compared with EIS test result. However, the sum R_o _+ R_CT _+ R_p_ is expected to be in agreement with the value measured by 0.1 Hz EIS signal. The sum of resistances R_o _+ R_CT_ + R_p_, i.e., the total resistance, from 10 sec pulse duration is 2.12 mΩ and 2.00 mΩ for discharge and charge respectively. The 0.1 Hz EIS result gives 1.91 mΩ (Fig. 5(b)). While these values are close (Fig. 7b), it is expected that the values from discharge pulse and charge pulse of longer than 5 sec will be higher compared to EIS because of the additional intercalation and de-intercalation above that associated with a pure DC load^29^. Charge transfer dynamics are relatively slower for an AC waveform because of the changing magnitude of current and signs. Furthermore, non-DC waveforms have been shown to circumvent lithium saturation at the electrode-electrolyte interface, thereby lowering the inhibition of ion transport attributed to polarisation and thus R_p_^37^. Therefore, the sum of R_o_ + R_CT_ + R_p_ from a 10 sec pulse duration is expected to be slightly higher than EIS results, as found in this study. In summary, it is suggested that the total resistance calculated from pulse power tests can be estimated directly from EIS test results.

The R_o_ value (1.62 mΩ) measured by the multisine signal test is much higher than that calculated by DC pulse and EIS methods. The maximum frequency applied in this technique was 1 Hz (Fig. 6). Inspection of the Bode plot shown in Fig. 5(b), indicates that at 1 Hz the cell has a resistance of 1.62 mΩ, which is exactly the value found in multisine signal test employing 1 Hz maximum frequency (Fig. 7b). In addition to the EIS test data, when the 1 second data point from a pulse duration is considered for a DC pulse, the resistance is found to be 1.61 mΩ for both charge and discharge (Fig. 7b). Therefore, the resistance R_o_ (which is a component in the 2^nd^ order ECM model), estimated by the multisine signal, employs a maximum frequency of 1 Hz, and thus cannot be labelled as a pure Ohmic resistance. Nevertheless, the resistance R_o_ estimated by the multisine technique exactly matches the resistance identified at 1 Hz from EIS. The total resistance (i.e., sum of R_o_ + R_CT_ + R_p_) for a multisine signal with 0.01 Hz minimum frequency is 2.83 mΩ, which is close to the resistance given by EIS at 0.01 Hz (Fig. 7b).

In conclusion, the results and subsequent analyses show that the resistance values measured by any technique are governed by the measurement timescale. As such, resistance measured by any technique can be estimated from the EIS test data, given its wide frequency content. Resistance values of any frequency corresponding to expected cell kinetics can therefore be obtained.

### Impact on application use

Battery resistance is used for different purposes, which include, for example, generating ECM parameter values, modelling and design of thermal management systems, ageing characterisation tests, SoH indication and more. It is an interesting open question as to which technique and value should be used for a particular application. ECMs, for example, are a well-established method of modelling the behaviour of Li-ion batteries. The model is reliant on resistance and resistances coupled with surface layer capacitance terms. Traditionally, pulse power data is used to determine the parameters of ECMs by fitting the model to the data and requiring that errors be minimised. The principal motivation for using pulse currents, in addition to the simplicity of the test method itself, is that high current pulses are assumed to mimic battery usage in real application scenarios. An inherent problem with parameter identification is the uniqueness of the solution, which gives rise to ambiguities between identified model parameters (in the simplest case R_o_ and R_CT_) and their actual physical values. This ambiguity is further enhanced when higher order equivalent circuit models utilized, which employ more RC circuits in order to describe more detailed physics such as the SEI or double layer (the latter often through constant phase elements). Given this ambiguity, the effectiveness of phenomenological models is judged solely on closeness of fit. Recently, it has been shown the pulse-multisine signals better represent actual battery usage and have a better fit to data^21^. In that regard, the pulse-multisine method can be a more effective way of defining resistance for systems modelling applications.

The 1 kHz resistance test, although is useful for a quick check in a manufacturing environment e.g. quality check, not much value is derived from it. Furthermore, whether the measured 1 kHz resistance value lies in the inductive or conductive region is highly dependent on the battery sample, therefore the single frequency (e.g. 1 kHz) needs to be chosen depending on the sample when employed for quality check.

In characterising batteries or characterising long term degradation, EIS may be a more appropriate method. This is because the method spans a large frequency range comprising various dynamics in the battery. So in addition to measuring internal resistance rise, conclusions can be derived for the contribution of SEI to degradation and other underlying mechanisms^29^. While EIS is a well-established technique^8^, the test duration for producing repeatable results^18^, the stringent requirements on the sensitive connections and other setup complexities, renders it cumbersome. However, as the evidence provided in this manuscript suggests, the resistance calculated from pulse current, multisine signal and 1 kHz impedance test can all be estimated from EIS test results to a good degree of accuracy; it may be advantageous therefore, to perform a reliable EIS test only.

## Conclusions

In this research, five different battery resistance measurement techniques were employed to measure resistance of a LiFePO_4_/C_6_ 20 Ah pouch cells. From comparison of the results, for the first time it has been shown that it is not the non-linearity of the lithium-ion battery, as suggested in other studies, rather the timescale associated with the technique itself that influences measured internal resistance. If the timescales at which the measurements are taken can be reconciled, the resulting values of resistance are comparable across the techniques. Furthermore, the discrepancy in measuring pure Ohmic resistance with different techniques originates from the limitation of the test equipment, where instead of the instantaneous voltage drop, a voltage drop in the timescale of charge-transfer was recorded. This is because a typical battery cycler has an upper limit of 10 Hz for measurement resolution.

For the first time, it is demonstrated that the resistance measured with different techniques can be estimated from an EIS test result. The root causes of discrepancies between the different methods as reported previously were identified and discussed. It is not possible to categorise a single best or correct technique; this will usually depend on the application and the availability of the test equipment, however, EIS can accurately provide separation and identification of all the individual resistance components, R_o_, R_CT_ and R_p_. In this manuscript, an in-depth analysis of the techniques was presented, this can assist researchers in deciding which techniques to use based on their application.

## References

[1] Aneke M, Wang M (2016). Energy storage technologies and real life applications – A state of the art review. Applied Energy.

[2] Wang J (2014). Degradation of lithium ion batteries employing graphite negatives and nickel–cobalt–manganese oxide + spinel manganese oxide positives: Part 1, aging mechanisms and life estimation. Journal of Power Sources.

[3] Li SE, Wang B, Peng H, Hu X (2014). An electrochemistry-based impedance model for lithium-ion batteries. Journal of Power Sources.

[4] Bruen T, Marco J (2016). Modelling and experimental evaluation of parallel connected lithium ion cells for an electric vehicle battery system. Journal of Power Sources.

[5] Robinson JB (2015). Detection of Internal Defects in Lithium-Ion Batteries Using Lock-in Thermography. ECS Electrochemistry Letters.

[6] Chacko S, Chung YM (2012). Thermal modelling of Li-ion polymer battery for electric vehicle drive cycles. Journal of Power Sources.

[7] Osswald PJ (2016). Current density distribution in cylindrical Li-Ion cells during impedance measurements. Journal of Power Sources.

[8] Waag W, Käbitz S, Sauer DU (2013). Experimental investigation of the lithium-ion battery impedance characteristic at various conditions and aging states and its influence on the application. Applied Energy.

[9] Ecker M (2012). Development of a lifetime prediction model for lithium-ion batteries based on extended accelerated aging test data. Journal of Power Sources.

[10] Aurbach D (2000). Review of selected electrode–solution interactions which determine the performance of Li and Li ion batteries. Journal of Power Sources.

[11] Beelen HPGJ, Raijmakers LHJ, Donkers MCF, Notten PHL, Bergveld HJ (2016). A comparison and accuracy analysis of impedance-based temperature estimation methods for Li-ion batteries. Applied Energy.

[12] Momma T, Matsunaga M, Mukoyama D, Osaka T (2012). Ac impedance analysis of lithium ion battery under temperature control. Journal of Power Sources.

[13] IEC 62660-1. Secondary lithium-ion cells for the propulsion of electric road vehicles – Part 1: Performance testing. 2012, International Electrotechnical Commission: Geneva, Switzerland.

[14] ISO. 12405-2:2012. Electrically propelled road vehicles - Test specification for lithium-ion traction battery packs and systems; Part 2: High-energy applications. 2012, British Standards Institution.

[15] United Sates Department of Energy. Battery Test Manual for Plug In Hybrid Electric Vehicles, V.T.P. Energy Efficiency and Renewable Energy, Editor. 2014: Idaho Operations Office.

[16] Uddin K, Picarelli A, Lyness C, Taylor N, Marco J (2014). An Acausal electro-thermal Li-ion battery models for automotive applications. Energies.

[17] Energizer. Battery internal resistance. data.energizer.com/PDFs/BatteryIR.pdf (Energizer, 2005).

[18] Barai A, Chouchelamane GH, Guo Y, McGordon A, Jennings P (2015). A study on the impact of lithium-ion cell relaxation on electrochemical impedance spectroscopy. Journal of Power Sources.

[19] Omar, N. *Assessment of rechargeable energy storage systems for plug-in hybrid electric vehicles* Ph.D. thesis, Vrije Universiteit Brussel, Brussel, Belgium, (2012).

[20] Widanage WD (2016). Design and use of multisine signals for Li-ion battery equivalent circuit modelling. Part 1: Signal design. Journal of Power Sources.

[21] Widanage WD (2016). Design and use of multisine signals for Li-ion battery equivalent circuit modelling. Part 2: Model estimation. Journal of Power Sources.

[22] Schweiger, H.-G. *et al*. Comparison of Several Methods for Determining the Internal Resistance of Lithium Ion Cells. *Sensors* **10**, 10.3390/s100605604 (2010). 10.3390/s100605604 PMC3247723 22219678

[23] Schuster E, Ziebert C, Melcher A, Rohde M, Seifert HJ (2015). Thermal behavior and electrochemical heat generation in a commercial 40 Ah lithium ion pouch cell. Journal of Power Sources.

[24] Nieto N (2013). Thermal Modeling of Large Format Lithium-Ion Cells. Journal of The Electrochemical Society.

[25] Kowal, J. *Spatially-resolved impedance of nonlinear inhomogeneous devices - using the example of lead-acid batteries* PhD thesis, RWTH Aachen University, (2010).

[26] Wang J (2011). Cycle-life model for graphite-LiFePO4 cells. Journal of Power Sources.

[27] Hu X, Li S, Peng H (2012). A comparative study of equivalent circuit models for Li-ion batteries. Journal of Power Sources.

[28] Smith K, Wang C-Y (2006). Solid-state diffusion limitations on pulse operation of a lithium ion cell for hybrid electric vehicles. Journal of Power Sources.

[29] Uddin K, Moore AD, Barai A, Marco J (2016). The effects of high frequency current ripple on electric vehicle battery performance. Applied Energy.

[30] Fuller TF, Doyle M, Newman J (1994). Relaxation Phenomena in Lithium‐Ion‐Insertion Cells. Journal of The Electrochemical Society.

[31] Gomez J, Nelson R, Kalu EE, Weatherspoon MH, Zheng JP (2011). Equivalent circuit model parameters of a high-power Li-ion battery: Thermal and state of charge effects. Journal of Power Sources.

[32] Barai, A. *Improvement of Consistency*, *Accuracy and Interpretation of Characterisation Test Techniques for Li-ion Battery cells for Automotive Application* Ph.D. thesis, University of Warwick, (2015).

[33] Plett GL (2004). Extended Kalman filtering for battery management systems of LiPB-based HEV battery packs: Part 2. Modeling and identification. Journal of Power Sources.

[34] Li J, Murphy E, Winnick J, Kohl PA (2001). The effects of pulse charging on cylcing characteristics of commercial lithium-ion batteries. Journal of Power Sources.

[35] Huria T, Ludovici G, Lutzemberger G (2014). State of charge estimation of high power lithium iron phosphate cells. Journal of Power Sources.

[36] Barsoukov, E. & Macdonald, J. R. *Impedance Spectroscopy*, *Theory*, *Experiment*, *and Applications*. 2 edn, (John Wiley & Sons, 2005).

[37] Purushothaman BK, Landau U (2006). Rapid Charging of Lithium-Ion Batteries Using Pulsed Currents. Journal of The Electrochemical Society.

